# Association between Child Nutritional Anthropometric Indices and Iron Deficiencies among Children Aged 6–59 Months in Nepal

**DOI:** 10.3390/nu16050698

**Published:** 2024-02-29

**Authors:** Kingsley Emwinyore Agho, Stanley Chitekwe, Sanjay Rijal, Naveen Paudyal, Sanjeev Kumar Sahani, Blessing Jaka Akombi-Inyang

**Affiliations:** 1 School of Health Sciences, Western Sydney University, Campbelltown, NSW 2560, Australia; k.agho@westernsydney.edu.au; 2 Faculty of Health Sciences, University of Johannesburg, Johannesburg 2094, South Africa; 3 Nutrition Section, United Nations Children’s Fund (UNICEF) Ethiopia, Addis Ababa 1169, Ethiopia; schitekwe@unicef.org; 4 United Nations Children’s Fund (UNICEF), Nepal Country Office P.O. Box 1187, United Nations (UN) House, Pulchowk, Kathmandu P.O. Box 107, Nepal; sarijal@unicef.org (S.R.); npaudyal@unicef.org (N.P.); ssahani@unicef.org (S.K.S.); 5 School of Population Health, University of New South Wales (UNSW), Sydney, NSW 2052, Australia

**Keywords:** anaemia, iron deficiency, ferritin, sTfR, children, stunting, wasting, underweight, Nepal

## Abstract

Developmental impairment remains an important public health problem among children in many developing countries, including Nepal. Iron deficiency in children may affect development and lead to anaemia. This study on 1702 children aged 6–59 months aimed to assess the association between nutritional anthropometric indices and iron deficiencies. Data for this study were extracted from the 2016 Nepal National Micronutrient Status Survey. Three nutritional anthropometric indices (stunting, wasting and underweight) and their association with anaemia and iron deficiencies (ferritin and sTfR biomarkers) were assessed by conducting multivariate statistical analyses. The prevalence of stunting, wasting and underweight among children aged 6–59 months was 35.6%, 11.7% and 29.0%, respectively. Most of the children were not stunted (64.4%), not wasted (71.0%) and not underweight (88.3%). Belonging to castes other than the Janajati, Dalit and Brahmin castes increased the odds of anaemia and iron deficiency (ferritin biomarker). Children in the age group 6–23 months were significantly at higher odds of having anaemia and iron deficiency (ferritin and sTfR biomarkers). Stunting significantly increased the odds of anaemia [adjusted odds ratio (OR): 1.55; 95% confidence interval (CI): (1.11, 2.17)], iron deficiency (ferritin biomarker [OR: 1.56; 95% CI: (1.16, 2.08)] and sTfR biomarker [OR: 1.60; 95% CI: (1.18, 2.15)]). Further, underweight significantly increased the odds of anaemia [OR: 1.69; 95% CI: (1.12, 2.54)] and iron deficiency (sTfR biomarker [OR: 1.48; 95% CI: (1.14, 1.93)]). Interventions to minimise the occurrence of anaemia and iron deficiencies among children in Nepal should focus on providing appropriate healthcare services that would reduce the burden of stunting and underweight.

## 1. Introduction

Anaemia is a medical condition in which the count of red blood cells or haemoglobin in the body is less than normal. Anaemia has been known as a major public health problem in low–middle-income countries (LMIC), including Nepal [1], with the subpopulations most affected being women of reproductive age, pregnant women and children [2]. It negatively affects a child’s cognitive development and physical growth from infancy to adolescence [3]. The most common causes of anaemia include parasitic infections and micronutrient deficiencies, particularly iron, vitamin A, folate, and vitamin B12 deficiencies [4,5,6,7]. In addition, malaria and genetic haemoglobin disorders are the main contributors to the anaemia burden [4]. Globally, anaemia affects about 43% of children [8], with approximately 40% of all children aged 6–59 months being anaemic due to iron deficiency [9].

Undernutrition is one of the main health issues highlighted in the 2015 Sustainable Development Goals (target 2.2: aspiring to end hunger by 2030) [10]. A 2015 report by the United Nations Children’s Emergency Fund (UNICEF) estimated that nearly 50% of deaths under 5 (about 3 million young lives a year) are attributed to undernutrition [11]. In Nepal, the burden of undernutrition and anaemia has been high over the years [12]. The 2016 Nepal Demographic and Health Survey reported that about 36% of children under 5 years of age were stunted, 10% were wasted, 27% were underweight and 53% were anaemic [12].

Iron deficiencies remain a major public health challenge, while the prevalence of undernutrition has increased over the past several decades [12]. The public health effects of iron deficiency include reduced work capacity and mental performance, poor growth development, impaired regulation of body temperature, impairments in behaviour and intellectual performance, and decreased resistance to infections [13,14]. Previous studies on the association between different indices of iron status and anthropometric measurements have reported that greater adiposity was associated with poorer iron status, which was demonstrated by lower serum iron (SI) and %transferrin saturation (%Tsat) and higher total iron-binding capacity (TIBC) and soluble transferrin receptor (sTfR) [15]. Overweight/obese children were also reported to require higher iron intake to maintain their iron homeostasis [15].

Currently, there are few studies on the association between undernutrition and different iron indices in children; knowledge of how anaemia and iron deficiency relate to nutritional anthropometric indices of children could enable public health professionals, the Nepalese government, and other stakeholders to identify and implement better evidence-based interventions to minimise the burden of these illnesses. To the best of our knowledge, there has not been any recent publication on iron deficiency and the nutritional status of children in Nepal. This present paper aims to assess the association between iron deficiency and nutritional anthropometric indices (stunting, wasting and underweight) among children aged 6–59 months in Nepal.

## 2. Methods

### 2.1. Study Design

Data used for this study were extracted from the 2016 Nepal National Micronutrient Status Survey (NNMSS). The NNMSS is a population-based study that uses a stratified multi-stage cluster sampling method. The country is divided into 15 strata, first by the five development regions (eastern, central, western, mid-western and far western) and then by three ecological zones (Terai, hill and mountain). The sample was selected using the 2011 census. Clusters (wards in the urban and rural areas) were used as the primary sampling units (PSUs). The minimum cluster size was defined as 100 households. Where necessary, clusters were combined prior to the first stage of sampling to meet this number. Further details of the sampling design and survey methodology are available in the 2016 NNMSS [16].

### 2.2. Study Participants

Nationally representative samples were collected on young children aged between 6 and 59 months.

### 2.3. Data Collection

The administered questionnaires were used to collect information on each household and for each child. Venous blood samples were collected from all the children. The young children’s questionnaire was used to collect information on socio-demographic characteristics from previous key national nutrition and other intervention programs in Nepal. The NNMS collected micronutrient supplementation intake (zinc, iron, folic acid, vitamin A, and multiple micronutrients), dietary diversity scale, two weeks recall of fever, cough, and diarrhoea, anthropometric measures, and documentation of the collection of biological data.

### 2.4. Anthropometric Measurements

Anthropometric measurements were collected from all of the participating children. For children aged less than 24 months, recumbent length was measured, and for those aged 24 months or older, standing height was measured using a standard height/length-measuring board (Shorr-Board). The weight of the participants was measured with an electronic SECA digital scale (UNICEF Electronic Scale or Uniscale). This scale makes room for the weighing of very young children through an automatic mother–child adjustment, which eliminates the weight of the mother while she is standing on the scale with her baby. The results of anthropometric measurements were immediately recorded on the questionnaire soon after each measurement.

The height-for-age, weight-for-height and weight-for-age indices of the children were calculated using growth standards published by the World Health Organization (WHO) in 2006. These growth standards were generated through data collected in the WHO Multicentre Growth Reference Study [17] and expressed in standard deviation units from the Multicentre Growth Reference Study median. The height-for-age, weight-for-height or weight-for-age index is an indicator of linear growth retardation and cumulative growth deficits in children.

### 2.5. Biological Indicators

Biological indicators assessed for the participating children included anaemia and iron status. The coverage for anaemia/haemoglobin was national, and it was assessed through a photometric method using the HemoCue^®^ Hemoglobin system on small blood samples. This method is satisfactorily accurate and precise in laboratory evaluations using standard methods. The recommended volume for this analysis is 10 μL. Coverage of iron deficiency/ferritin was also national and in the five regions as well as in the three ecological zones. Ferritin is recommended by the World Health Organisation (WHO) as an indicator of iron deficiency in populations and is a measure of iron stores. Ferritin is an acute-phase reactant protein and is influenced by inflammation and infections. It is analysed at low cost using the enzyme-linked immunosorbent assay (ELISA) method. Soluble transferrin receptor (sTfR) is an indicator of iron insufficiency when iron stores are depleted (and assuming the absence of other causes of abnormal erythropoiesis). It can be elevated by thalassemia and is thought to be less influenced by inflammation and infection than ferritin. It can also be analysed at low cost using the ELISA method. The recommended volume for analysis for both ferritin and sTfR is 30 μL.

### 2.6. Specimen Collection and Processing

Blood samples were collected from the participating children to assess their micronutrient status. Blood was collected at the time of the survey. Details of the specimen collection can be obtained from the NNMSS final report [16].

### 2.7. Study Outcomes

The outcome variables for this study were anaemia and iron deficiency (ferritin and sTfR). Anaemia is one of the diseases that result due to low levels of haemoglobin in the blood. For children aged 6–59 months, anaemia is diagnosed when the haemoglobin level is less than 11.0 g/dL. Ferritin and sTfR are biomarkers for determining the deficiency of iron in the blood. In this study, both ferritin and sTfR were BRINDA adjusted. For all age groups, the sTfR level needs to be greater than 8.3 mg/L. Each occurrence of anaemia or iron deficiency was coded as 1; the non-occurrence of anaemia or iron deficiency was coded as 0.

## 3. Exposure Variables

### 3.1. Stunting (Height-for-Age)

Children with height-for-age Z-score below minus two standard deviations (−2 SD) from the median of the WHO reference population are stunted or chronically malnourished [17].

### 3.2. Wasting (Weight-for-Height)

Children with weight-for-height Z-scores below minus two standard deviations (−2 SD) from the median of the WHO reference population are regarded as wasted or acutely malnourished [17].

### 3.3. Underweight (Weight-for-Age)

Children whose weight-for-age is below minus two standard deviations (−2 SD) from the WHO Multicentre Growth Reference Study median [6] are classified as underweight [17].

The exposure variables were coded as 1 if stunted, wasted or underweight; otherwise, they were coded as 0.

### 3.4. Potential Confounding Variables

The selection of possible confounding variables was informed by the extant literature on factors associated with micronutrient deficiency [18,19,20,21], as well as their availability in the NNMSS dataset. These variables were categorised into individual-level factors, household-level factors, community-level factors, anthropometric factors, health status factors and diet habits (a day prior to the survey).

The individual-level factors included the child’s age, gender and relationship to the carer; household-level factors included the household wealth index and ethnicity. The household wealth index was represented as a score of household assets through the principal components analysis (PCA) method [22]. The index was categorised into three classes, namely, poor, middle and rich. The bottom 40% of the households were referred to as the poor; the next bottom 40% was referred to as the middle, and the top 20% was referred to as the rich.

Community-level factors included geographical region and ecological zone. Anthropometric factors included the child’s stunting, wasting and underweight status; health status factors included the contraction of fever, cough and diarrhoea; diet habit factors included the participants’ consumption of the different food groups and food security. Water and sanitation factors included quality of drinking water, type of toilet facility used in participants’ households and their water treatment habits.

### 3.5. Statistical Analysis

In this study, we used STATA/MP version 14 (Stata Corp., College Station, TX, USA) to conduct all statistical analyses. The ‘Svy’ commands were employed to allow for adjustments for the cluster-sampling design and weight. We first conducted frequency tabulations for exposure and all confounding factors, and this was followed by prevalence and univariate analyses that independently examined anaemia, ferritin and sTfR via the nutritional anthropometric index. Multivariate analyses were used to examine the association between child nutritional anthropometric indices and iron deficiencies. As part of the multivariate analyses, a staged modelling technique was carried out.

As a process of multivariate modelling technique, all community and household level factors were first entered into the baseline multivariable model with a backward elimination process to remove statistically non-significant variables (Model 1). In the next stage, individual-level factors were examined with model 1 (Model 2). Next, health-related factors were assessed with model 2 (Model 3). In the fourth modelling stage, water and sanitation factors were examined with model 3 (Model 4). In the final model (Model 5), the exposure variables (stunting, wasting and underweight) were examined with those variables significant in models 1–4.

In the final model, we tested and reported any co-linearity. The odds ratios with 95% confidence intervals were calculated to assess the adjusted odds of independence.

## 4. Results

### 4.1. Characteristics of the Sample

The characteristics of the study population are summarised in Table 1. Of the 1702 children, more than two-thirds were aged 24–59 months. Male children were 53.5%, while females were 46.5%. A large proportion of the children (96.7%) lived with their biological parents. In the two weeks preceding the survey, most of the children did not have fever, cough, or diarrhoea (61.6%, 61.8% and 80.4%, respectively). The majority of participants were not stunted, not wasted and not overweight (64%, 71% and 88%, respectively). Less than one in every five children (19%) were from rich households, and more than three-quarters of the children (87%) resided in rural areas. The central region was home to most of the children (36.5%), and more than one-half (~51%) of them were from the Terai ecological zone. Less than one-third of the children (~23%) consumed food from less than four food groups, and approximately 8% came from households with severe food insecurity and unimproved sources of drinking water. Most of the children were from households which drank untreated water (83.4%) and that had flush or pour-flush toilets (~71%).

### 4.2. Prevalence of Anaemia and Iron Deficiency by Nutritional Anthropometric Index

#### 4.2.1. Anaemia

Prevalence and adjusted odds ratios (aOR) of anaemia, ferritin and sTfR in terms of the nutritional anthropometric index of the children are summarised in Table 2. Approximately 23% [95% confidence interval (CI): (18.3, 28.2)] of stunted children were anaemic, whilst 17.1% [95% CI: (13.9, 20.7)] who were not stunted had anaemia. The likelihood of developing anaemia was significantly higher among stunted children compared with those who were not stunted [aOR: 1.44; 95% CI: (1.04, 1.98)].

Approximately 23% of the children who were wasted were anaemic [95% CI: (15.6, 32.9)], whilst 18.6% of those not wasted had anaemia [95% CI: (15.4, 22.2)]. Children who were wasted were significantly more predisposed to anaemia compared to those who were not wasted [aOR: 1.32; 95% CI: (0.78, 2.21)], although this was not statistically significant.

More than 24% of the underweight children had anaemia, whilst approximately 17% did not have the illness [95% CI: (13.9, 20.6)]. The likelihood of contracting anaemia was significantly higher among underweight children compared with those who were not underweight [aOR: 1.58; 95% CI: (1.11, 2.26)]. The prevalence of undernutrition due to zinc deficiency was evenly distributed, ranging from 20% to 24%, and no statistical difference was observed (Table 2).

#### 4.2.2. Iron Deficiency (Ferritin Biomarker)

Slightly more than 40% of the stunted children were iron deficient (ferritin biomarker) [95% CI: (33.2, 45.7)], whilst 33.1% of those not stunted were iron deficient (ferritin biomarker). Stunted children were significantly more prone to being iron deficient (ferritin biomarker) compared with those who were not stunted [aOR: 1.35; 95% CI: (1.04, 1.77)].

Approximately 39% of children who were wasted were iron deficient (ferritin biomarker) [CI: (29.4, 49.8)]. Close to 38% of children who were underweight were deficient in iron (ferritin biomarker) [95% CI: (31.0, 44.9)], whilst 34.7% of those who were not underweight had iron deficiency (ferritin biomarker) [CI: (30.8, 38.8)].

#### 4.2.3. Iron Deficiency (sTfR Biomarker)

Approximately 40% of the stunted children were deficient in iron (sTfR biomarker) [CI: (33.0, 48.0)], and 34.8% of those who were not stunted were not iron deficient (sTfR biomarker).

Children who were wasted and those not wasted who developed iron deficiency (sTfR biomarker) were almost equally represented [37.3%, 95% CI: (29.1, 46.3) and 36.8%, 95% CI: (31.8, 42.0), respectively]. Approximately 41% of the underweight children were anaemic [CI: (34.3, 48.7)] against almost 35% of those who were not underweight being anaemic [CI: (30.2, 39.9)].

#### 4.2.4. Association of Nutritional Anthropometric Indices with Anaemia and Iron Deficiencies

**Figure 1:**
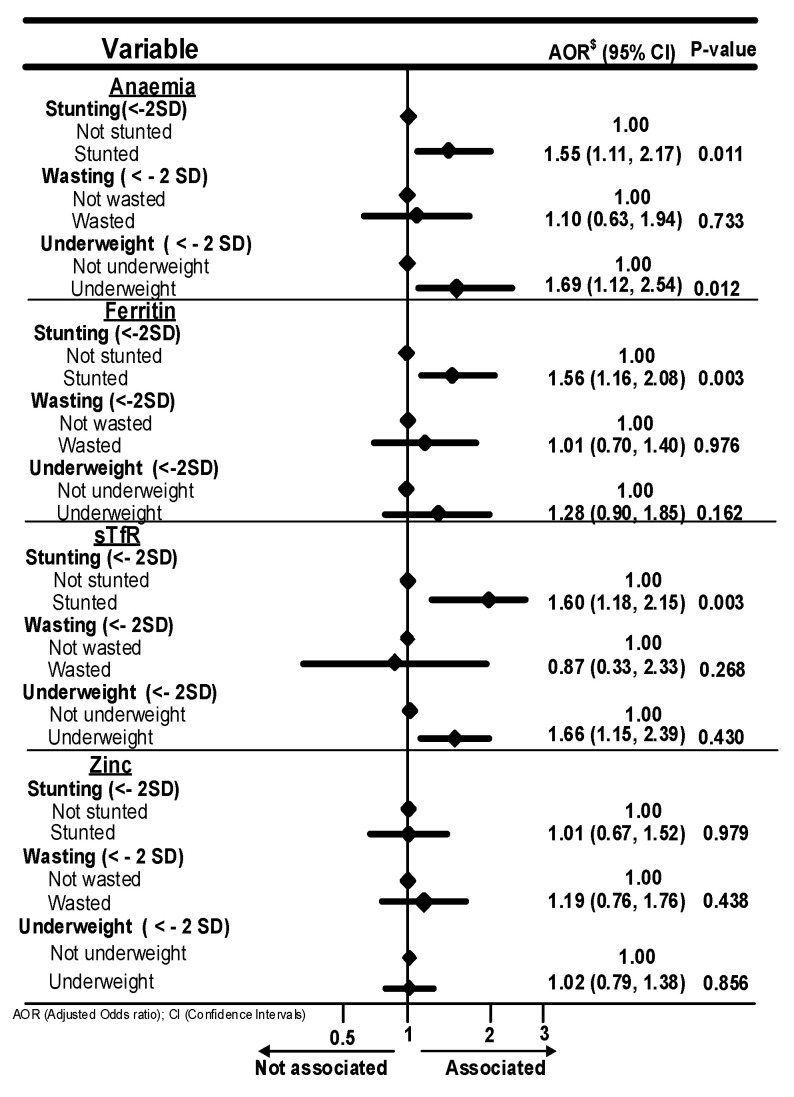
Association of nutritional anthropometric indices with anaemia, iron and zinc deficiencies. $: Independent variables adjusted are: Community level, household level, Individual level factors, health status, current breastfeeding, anthropometric and nutrition, Water and Sanitation factors.

Figure 1 summarises the association of nutritional anthropometric indices with anaemia, iron deficiencies and zinc deficiency. Stunting [aOR: 1.55; 95% CI: (1.11, 2.17)] and underweight [aOR: 1.69; 95% CI: (1.12, 2.54)] significantly increased the likelihood of anaemia in the children. Stunting significantly increased the odds of iron deficiency (ferritin biomarker) ([aOR: 1.56; 95% CI: (1.16, 2.08)]). The odds of iron deficiency (sTfR biomarker) were increased by both stunting [aOR: 1.60; 95% CI: (1.18, 2.15)] and underweight [aOR: 1.48; 95% CI: (1.14, 1.93)]. The odds of stunting, wasting and underweight among children aged 6–59 months with zinc deficiency were 1% (aOR: 1.01; 95% CI: (067, 1.52, *p* = 0.979), 19% (aOR: 1.19; 95% CI: (0.76, 1.76, *p* = 0.438), and 2% (aOR: 1.02; 95% CI: (0.79, 1.38, *p* = 0.856), respectively.

After adjusting for community, household and individual-level factors, anthropometry and nutrition factors, health status, and water and sanitation factors, significant factors associated with anaemia and iron deficiencies are summarised in Table 3. Children from castes other than the Janajati and Dalit castes had an increased risk of anaemia and iron deficiency (ferritin biomarker). Younger children aged 6–23 months had increased odds of anaemia and iron deficiency (ferritin and sTfR biomarkers). Children who had a fever in the two weeks prior to the survey had increased odds of iron deficiency (ferritin biomarker), and those from the Terai ecological zone had increased odds of iron deficiency (sTfR biomarker). Further, children who did not have diarrhoea in the two weeks preceding the survey had decreased odds of iron deficiency (ferritin biomarker), and those who lived in households with treated water had decreased odds of anaemia. Children from households with severe food insecurity had increased odds of iron deficiency (sTfR biomarker). Children aged 6–59 months living in rural areas have higher odds of zinc deficiency compared to children 6–59 months living in urban areas.

## 5. Discussion

Based on the analysis of the NNMSS dataset, this current study revealed that more Nepalese children aged 6–59 months who were stunted, wasted and underweight had anaemia and iron deficiencies (both ferritin and sTfR biomarkers) compared to their counterparts who were not. Specifically, stunting was positively associated with iron deficiencies, while being underweight was associated with anaemia and sTfR biomarkers.

Our study revealed that children who were stunted were at increased odds of developing anaemia and iron deficiencies (ferritin and sTfR biomarkers). Similar findings have been reported in previous studies. For instance, a study to determine if stunting could have an effect on the severity of malaria-associated anaemia in African children reported that children who were stunted were at an increased risk of severe anaemia and high serum concentrations of C-reactive protein and sTfR [23]. Another study on polyparasite helminth infections and their association with anaemia and undernutrition in northern Rwanda reported that stunted relatives of non-stunted children were found to be much more inclined to have anaemia [24]. On the other hand, research has supported the assertion that deficiency in iron, which leads to anaemia, is a contributing factor to poor growth. It has also been reported that iron supplementation to anaemic children has a positive effect on linear growth [25].

In this study, children aged between 6 and 59 months belonging to the Janajati caste had lower odds of ferritin than those from the Brahmin/Chettri caste. This finding may be attributed to the fact that a considerable number of women and children from the Janajati caste belong to the lowest wealth quintile. The relationship between caste, wealth and suboptimal nutrition has been reported in previous studies [26]. Furthermore, in the univariable logistic regression analysis, anaemia and sTfR biomarkers were associated with current breastfeeding. However, this association was no longer significant after adjusting for potential confounding factors such as diarrhoea (2 weeks prior to the survey), child’s age (6–59 months) and household food security. These findings supported a cross-sectional study conducted in Kenya among 6–10-month-old infants, which found that duration of breastfeeding correlated haemoglobin and sTfR concentrations and concluded that sTfR concentrations were lower among infants who were still exclusively breastfed at 6–10 months [27].

We found that children who were underweight had an increased likelihood of developing anaemia (low haemoglobin levels). This finding is consistent with findings from previous research on the risk factors for anaemia among school children in the Tanga region, Tanzania [28]. The study found that the risk of all forms of anaemia was high among underweight children, among other factors such as iron deficiency, ascariasis, schistosomiasis, and vitamin A deficiency. The increased odds of experiencing lower haemoglobin levels among underweight children could suggest that the observed anaemia may be diet-related.

In this study, younger children had increased odds of developing anaemia and iron deficiency, which is consistent with a finding from a past study in Rwanda, which reported that older children were significantly less prone to anaemia than younger children [24]. A similar finding was reported in a past study in Kenya [29] and other West African countries [30]. This finding may be attributed to the fact that younger children experience a higher demand for iron, which is associated with early childhood development. Extra iron is therefore required during the growing period of children to increase muscle mass and for the growth spurt of adolescence. The lack of essential minerals and vitamins in the diet of these children, or when such micronutrients are marginalized, may result in nutritional deficiencies.

Our study also reported that children from the Terai ecological zone were more prone to iron deficiency (sTfR levels), while children from castes other than the Janajati, Dalit and Brahmin/Chettri had increased odds of developing anaemia and iron deficiency (ferritin biomarker). Such differences have been found in other past studies [20,31,32,33]. Similar findings have been reported in previous studies, which indicate that geographical location is associated with iron deficiency and is often higher in rural areas than in urban areas [3,34]. A Ugandan study reported the prevalence of anaemia to be higher in one sector (the Bulange sub-county) than in other areas (the Namutumba and Magada sub-counties) [35]. The differences may be a manifestation of some regions or sectors being better off in terms of nutrition education, availability of appropriate and adequate foods or socio-economic resources.

In this study, children aged 6–59 months in rural Nepal were found to have a significantly increased likelihood of zinc deficiency. Several factors, including socio-economic, environmental, and healthcare-related factors, may contribute to this difference in zinc deficiency prevalence between rural and urban areas. In rural settings, limited access to diverse and nutritious foods, poor sanitation and hygiene practices and lower socio-economic status may contribute to inadequate zinc intake and absorption. Additionally, rural populations may have limited access to healthcare services and nutritional interventions compared to urban areas, further exacerbating the risk of nutrient deficiencies. Addressing this rural–urban disparity in the prevalence of zinc deficiency in Nepal would require targeted interventions to improve access to nutritious foods, healthcare services and public health initiatives in rural communities to ensure adequate zinc intake and reduce the burden of zinc deficiency among children. This observation aligns with the results of a cross-sectional study conducted in Nepal, which reported that zinc deficiency was approximately three times more prevalent among children aged 6–59 months residing in rural areas compared to their urban counterparts [36]. These findings are also in line with research conducted in Ethiopia [37] and rural Benin [38]. A key strength of this current study was that the NNMSS, from which our data were acquired, is population-based and nationally representative with large datasets. Consequently, the findings of the study may be applied to any section of the country. The study, however, was limited in a number of ways, which need to be considered when interpreting the results. First, because the study was cross-sectional in design, the causation between anaemia and iron deficiency and their associated factors could not be established. Second, biomarkers such as ferritin and sTfR may not necessarily represent the amount of iron obtained from foods and may be influenced by inflammation, multi-morbidity and interactions between nutrients and drugs.

## 6. Recommendations

The Ministry of Health and Population and other stakeholders in Nepal should intensify their efforts to institute appropriate education about adequate complementary feeding for young children. Providing information on indigenous Nepalese foods that are iron-rich could go a long way in reducing the prevalence of anaemia in the country.

Since our study found stunting to be significantly associated with anaemia and iron deficiency, a strategic way to minimise anaemia and iron deficiency by the government and other stakeholders would be to pay attention to the factors that increase the risk of stunting, such as being male [39,40,41,42,43], household poverty [39,43,44,45] and baby delivery with assistance from untrained personnel [46,47].

## 7. Conclusions

In this present study, we demonstrated the association between nutritional anthropometric indices and anaemia and iron deficiency among children aged 6–59 months. Stunted children were more likely to develop anaemia and iron deficiencies (both ferritin and sTfR biomarkers), while underweight children were found to have an increased risk of developing anaemia. The Ministry of Health and Population in Nepal and other stakeholders should intensify efforts to minimise anaemia and iron deficiency among children aged 6–59 months. Factors associated with child stunting and underweight should be considered, most importantly infant and young child feeding practices, which consist mainly of appropriate breastfeeding and complementary feeding.

## Figures and Tables

**Table 1:** Characteristics distribution of children aged 6–59 months.

Characteristics	N (%)
**Individual level factors**	
Child’s age (months)	
6–23	520 (30.6)
24–59	1180 (69.4)
Child’s gender	
Male	909 (53.5)
Female	791 (46.5)
***Relation to child***	
Biological parents	1643 (96.7)
Others	57 (3.3)
***Health status***	
Child had fever	
Yes	619 (36.4)
No	1080 (61.6)
Child had cough	
Yes	649 (38.2)
No	1051 (61.8)
Child had diarrhoea	
Yes	334 (19.6)
No	1366 (80.4)
**Anthropometry and nutrition**	
Current Breastfeeding	
No	1229 (71.9)
Yes	481 (28.1)
Child Stunted (<−2 SD)	
No	1095 (64.4)
Yes	605 (35.6)
Child wasted (<−2 SD)	
No	1495 (88.3)
Yes	199 (11.7)
Child underweight (<−2 SD)	
No	1206 (71.0)
Yes	494 (29.0)
**Household level factors**	
Household Wealth Index	
Poor	698 (41.1)
Middle	676 (39.8)
Rich	325 (19.1)
Ethnicity (Caste)	
Brahmin/Chettri	518 (30.5)
Dalit	313 (18.4)
Janajati	496 (29.2)
Others	371 (21.9)
**Community level factors**	
Residence	
Urban	220 (13.0)
Rural	1480 (87.0)
Geographical region	
Eastern	369 (21.6)
Central	620 (36.5)
Western	297 (17.5)
Mid-western	242 (14.3)
Far-western	171 (10.1)
Ecological zone	
Mountain	130 (7.6)
Hill	709 (41.7)
Terai	861 (50.7)
Dietary Diversity	
<4 foods	1304 (76.7)
4 or more foods Household food security	395 (23.3)
Food security	909 (53.5)
Mild food security	183 (10.8)
Moderate food security	464 (27.3)
Severe food insecurity	143 (8.4)
**Water and Sanitation**	
Source of drinking water	
Improved	1564 (92.0)
Unimproved	136 (8.0)
Type of toilet facility	
Flush or Pour flush toilet	1210 (71.2)
Pit Latrine	490 (28.8)
Water treatment habit	
Yes	282 (16.6)
No	1417 (83.4)

**Table 2:** Prevalence and 95% confidence interval (CI) and unadjusted odd ratios (OR) of anaemia, ferritin and sTfR by nutritional anthropometric index among children aged 6–59 months.

Variable	Prevalence (95%CI)	OR (95% CI)	*p*-Value
**Anaemia (Haemoglobin < 11.0 g/dL)**			
**Stunting (<−2 SD)**			
Not stunted	17.1 (13.9, 20.7)	1.00	
Stunted	22.8 (18.3, 28.2)	1.44 (1.04, 1.98)	0.027
**Wasting (<−2 SD)**			
Not wasted	16.9 (13.9, 20.6)	1.00	
Wasted	24.4 (19.2, 30.5)	1.58 (1.11, 2.26)	0.012
**Underweight (<−2 SD)**			
Not underweight	18.6 (15.4, 22.2)	1.00	
Underweight	23.1 (15.6, 32.9)	1.32 (0.78, 2.21)	0.294
**BRINDA adjusted Ferritin (<15 μg/L)**			
**Stunting (<−2 SD)**			
Not stunted	33.1 (29.7, 36.7)	1.00	
Stunted	40.1 (33.2, 47.5)	1.35 (1.04, 1.77)	0.026
**Wasting (<−2 SD)**			
Not wasted	34.7 (30.8, 38.8)	1.00	
Wasted	37.7 (31.0, 44.9)	1.14 (0.87, 1.49)	0.328
**Underweight (<−2 SD)**			
Not underweight	35.2 (31.2, 39.3)	1.00	
Underweight	39.2 (29.4, 49.8)	1.19 (0.80, 1.77)	0.397
**BRINDA adjusted sTfR (>8.3 mg/L)**			
**Stunting (<−2 SD)**			
Not stunted	34.8 (30.5, 39.4)	1.00	
Stunted	40.3 (33.0, 48.0)	1.26 (0.96, 1.66)	0.089
**Wasting (<−2 SD)**			
Not wasted	34.9 (30.2, 39.9)	1.00	
Wasted	41.3 (34.3, 48.7)	1.31 (0.99, 1.73)	0.054
**Underweight (<−2 SD)**			
Not underweight	36.8 (31.8, 42.0)	1.00	
Underweight	37.3 (29.1, 46.3)	1.02 (0.70, 1.49)	0.906
**Zinc deficiency (adjusted serum zinc < 65.0 μg/dL)**			
**Stunting (<−2 SD)**			
Not stunted	20.3 (16.5, 24.7)	1.00	
Stunted	20.8 (15.9, 26.8)	1.03 (0.70, 1.51)	0.874
**Wasting (<−2 SD)**			
Not wasted	20.0 (16.9, 23.6)	1.00	
Wasted	24.2 (16.3, 34.2)	1.27 (0.81, 1.99)	0.293
**Underweight (<−2 SD)**			
Not underweight	20.1 (16.6, 24.1)	1.00	
Underweight	21.3 (17.0, 26.3)	1.08 (0.83, 1.40)	0.579

**Table 3:** Non-biological factors associated with anaemia, ferritin, sTfR and zinc deficiency among children aged 6–59 months in Nepal: adjusted odd ratios (AOR); CI: confidence interval.

Characteristics	Adjusted OR (95% CI)	*p*-Value	Adjusted OR (95% CI)	*p*-Value	Adjusted OR (95% CI)	*p*-Value	Adjusted OR (95% CI)	*p*-Value
	Anaemia		Ferritin		sTfR		Zinc	
**Ecological zone**								
Mountain					1.00			
Hill					1.39 (0.86, 2.25)	0.170		
Terai					3.80 (2.20, 6.59)	<0.001		
**Place of residence**								
Urban							1.00	
Rural							3.03 (1.56, 5.92)	0.001
**Ethnicity (Caste)**								
Brahmin/chettri	1.00		1.00					
Dalit	1.13 (0.70, 1.86)	0.595	0.96 (0.61, 1.52)	0.859				
Janajati	1.20 (0.76, 1.88)	0.425	0.50 (0.32, 0.77)	0.002				
Others	2.60 (1.65, 4.10)	<0.001	1.51 (1.08, 2.13)	0.019				
**Child Age**								
24–59 months	1.00		1.00		1.00			
6–23 months	3.26 (2.43, 4.38)	<0.001	4.18 (3.00, 5.83)	<0.001	4.39 (3.39, 5.95)	<0.001		
Child had diarrhoea								
Yes	1.00		1.00					
No	0.61 (0.38, 0.97)	0.036	0.63 (0.46, 0.86)	0.004				
Child’s household treated water								
No	1.00							
Yes	0.53 (0.34, 0.83)	0.007						
Child had fever								
No			1.00					
Yes			1.83 (1.30, 0.57)	0.001				
**Household wealth index**								
Food secure					1.00			
Mild food security					0.88 (0.53, 1.39)	0.540		
Moderate food security					1.24 (0.86, 1.80)	0.244		
Severe food insecurity					1.41 (1.00, 1.99)	0.048		

## Data Availability

The study was based on an analysis of existing survey datasets that are available by applying to UNICEF Nepal, with all identifier information removed. Written informed consent for the present analysis was not necessary because secondary data analysis did not involve interaction with the participants. The data collection methods for the Nepal National Micronutrient Status Survey Report 2016 used in this analysis, including the consent process, have been previously described at [16]. Written informed consent for the present analysis was unnecessary because secondary data analysis did not involve interaction with the participants.
